# Improvements in malaria surveillance through the electronic Integrated Disease Surveillance and Response (eIDSR) system in mainland Tanzania, 2013–2021

**DOI:** 10.1186/s12936-022-04353-w

**Published:** 2022-11-08

**Authors:** Joseph J. Joseph, Humphrey R. Mkali, Erik J. Reaves, Osia S. Mwaipape, Ally Mohamed, Samwel N. Lazaro, Sijenunu Aaron, Frank Chacky, Anna Mahendeka, Hermes S. Rulagirwa, Mwendwa Mwenesi, Elibariki Mwakapeje, Ally Y. Ally, Chonge Kitojo, Naomi Serbantez, Ssanyu Nyinondi, Shabbir M. Lalji, Ritha Wilillo, Abdul-wahid Al-mafazy, Bilali I. Kabula, Claud John, Donal Bisanzio, Erin Eckert, Richard Reithinger, Jeremiah M. Ngondi

**Affiliations:** 1 RTI International, Dar es Salaam, United Republic of Tanzania; 2 U.S. President’s Malaria Initiative, U.S. Center for Disease Control and Prevention, Dar es Salaam, United Republic of Tanzania; 3 Ministry of Health, Dodoma, United Republic of Tanzania; 4 National Malaria Control Programme, Dodoma, United Republic of Tanzania; 5 U.S. President’s Malaria Initiative, U.S. Agency for International Development, Dar es Salaam, United Republic of Tanzania; 6 World Health Organization, Dar es Salaam, Tanzania; 7 RTI International, Washington, DC USA

**Keywords:** Malaria surveillance, eIDSR, Weekly reporting, Monthly reporting, Timeliness, DHIS2

## Abstract

**Background:**

Tanzania has made remarkable progress in reducing malaria burden and aims to transition from malaria control to sub-national elimination. In 2013, electronic weekly and monthly reporting platforms using the District Health Information System 2 (DHIS2) were introduced. Weekly reporting was implemented through the mobile phone-based Integrated Disease Surveillance and Response (eIDSR) platform and progressively scaled-up from 67 to 7471 (100%) public and private health facilities between 2013 and 2020. This study describes the roll-out and large-scale implementation of eIDSR and compares the consistency between weekly eIDSR and monthly DHIS2 malaria indicator data reporting, including an assessment of its usefulness for malaria outbreak detection and case-based surveillance (CBS) in low transmission areas.

**Methods:**

The indicators included in the analysis were number of patients tested for malaria, number of confirmed malaria cases, and clinical cases (treated presumptively for malaria). The analysis described the time trends of reporting, testing, test positivity, and malaria cases between 2013 and 2021. For both weekly eIDSR and monthly DHIS2 data, comparisons of annual reporting completeness, malaria cases and annualized incidence were performed for 2020 and 2021; additionally, comparisons were stratified by malaria epidemiological strata (parasite prevalence: very low < 1%, low 1 ≤ 5%, moderate 5 ≤ 30%, and high > 30%).

**Results:**

Weekly eIDSR reporting completeness steadily improved over time, with completeness being 90.2% in 2020 and 93.9% in 2021; conversely, monthly DHIS2 reporting completeness was 98.9% and 98.7% in 2020 and 2021, respectively. Weekly eIDSR reporting completeness and timeliness were highest in the very low epidemiological stratum. Annualized malaria incidence as reported by weekly eIDSR was 17.5% and 12.4% lower than reported by monthly DHIS2 in 2020 and 2021; for both 2020 and 2021, annualized incidence was similar across weekly and monthly data in the very low stratum.

**Conclusion:**

The concurrence of annualized weekly eIDSR and monthly DHIS2 reporting completeness, malaria cases and incidence in very low strata suggests that eIDSR could be useful tool for early outbreak detection, and the eIDSR platform could reliably be expanded by adding more indicators and modules for CBS in the very low epidemiological stratum.

## Background

Tanzania has made remarkable progress in reducing its malaria burden by scaling up malaria prevention and control interventions, including malaria rapid diagnostic tests (RDT), artemisinin-based combination therapy (ACT), long-lasting insecticidal nets (LLINs), indoor residual spraying (IRS) of households with insecticide, social and behaviour change (SBC), and malaria surveillance [1]. As a result, malaria prevalence in children under five years of age has decreased nationally from 17% in 2007 to 7% in 2017 [2, 3].

The World Health Organization (WHO) advocates for malaria surveillance systems to be tailored to various epidemiologic settings. In 2012 and 2018, WHO released operational manuals for malaria surveillance to provide guidance to country settings that are in the control and/or elimination phases [4–6]. In the control phase, the objective of malaria programmes is tailored towards reducing malaria incidence and mortality as rapidly and cost-effectively as possible. In addition, Pillar 3 of the Global Technical Strategy for Malaria 2016–2030 (GTS) recognizes the importance of malaria surveillance and elevates surveillance to be a core intervention [7]. Robust malaria surveillance systems support National Malaria Control Programmes (NMCP) in their strategic decision making, including targeting interventions more effectively as they progress towards elimination, as well as prevent re-establishment of transmission where malaria has already been eliminated [6, 7]. Consistent with the above WHO recommendations, starting in 2012, the NMCP of mainland Tanzania developed a surveillance platform capable of monitoring malaria trends and detecting sudden increases in transmission more accurately and timely, especially in the geographic Lake Zone around Lake Victoria, where the implementation of multiple interventions had resulted in a substantial reduction in malaria burden [2, 3] The two main elements of this surveillance platform are the country’s health management information system (HMIS), which is based on the District Health Information Software-2 (DHIS2) platform [8–11], and the electronic Integrated Diseases Surveillance and Response (eIDSR) system [10–13].

In 2018, the NMCP stratified mainland Tanzania into four epidemiological strata according to malaria endemicity: “very low”, “low”, “moderate”, and “high” [14]. This stratification was included in the NMCP’s Supplementary Malaria Mid-term Strategic Plan (SMMSP) 2018–2020 [15], which recommended targeting and implementing programmatic approaches and interventions to the district level in alignment with these strata. In the SMMSP, the NMCP also emphasized the need to integrate vector control and case management interventions with efforts to enhance malaria surveillance, particularly in unstable malaria transmission areas, to mitigate the risk of malaria outbreaks and malaria resurgence. Such an approach would also enable districts to develop the capacity to swiftly respond after an outbreak has been detected and to effectively contain outbreaks within two weeks as per national guidelines [16]. In addition, the SMMSP also advocated for enhanced surveillance in the “very low” epidemiological stratum, including use of approaches such as active case detection (ACD), reactive case detection (RCD), and case notification and follow-up (case-based surveillance, CBS) [5, 17].

The aims of the study presented here were to: (1) describe the implementation of weekly malaria reporting through eIDSR; (2) compare the completeness and timeliness between the weekly eIDSR and monthly DHIS2 reporting; (3) assess the consistency of weekly eIDSR data compared to monthly DHIS2 data by epidemiological strata; and (4) assess usefulness of eIDSR for malaria outbreak detection and CBS in low transmission areas.

## Methods

### Monthly reporting through DHIS2 platform

DHIS2 is an open-source web-based software platform for the electronic reporting, analysis, and dissemination of health programmatic data [18], which can be accessed by authorized health policy makers, managers and care providers at all levels of the health care delivery system [9]. Typically, using DHIS2, health facility data is captured at district level, before being reported to regional and national levels; the design, roll-out and implementation of DHIS2 in mainland Tanzania has been fully described [10]. Tanzania migrated from the previous paper-based reporting system to DHIS2 between 2009 and 2011, when the Ministry of Health (MOH) executed a monitoring and evaluation (M and E) strengthening initiative to improve the country's HMIS [11]. By the end of December 2013, the DHIS2 platform had been rolled out in all 26 regions of mainland Tanzania [19].

Health service delivery data are primarily generated by health facilities through the provision of services such as antenatal care (ANC), labour and delivery, outpatient consultations [i.e., Outpatient Department (OPD)], and inpatient admissions [Inpatient Department (IPD)]. Specifically, for malaria, when suspected malaria patients are seen by facility service providers, they are—depending on the service provided—recorded in respective registers [i.e. HMIS register book #5(OPD), IPD, HMIS register book #7(ANC) and HMIS register book #10 (LAB)] [19]. The core malaria diagnosis indicators, aggregated by age group (< 1 month, 1 month to < 1 year, 1 year to < 5 years, 5 years to < 60 years, > 60 years) and gender, include: number of people tested for malaria by microscopy or RDT; number of patients with a positive test for malaria infection; and number of clinical malaria cases treated presumptively. At the end of each day, the information in the OPD register is summarized on paper-based tally sheets, which are then used to compile monthly paper-based summary forms for submission to the District Health Office. At the district level, data from these paper-based monthly summary forms are entered into DHIS2 through a web-based portal. Subsequently, data are then accessed by users at sub-national and national levels for processing, analysis, decision making, as well as feedback to lower levels health officials for action. Previous month’s data should be submitted into DHIS2 by the 15th day of any given month and data submitted after this data are considered as late reporting. In this manuscript, malaria data that are reported monthly are referred to as “monthly DHIS2”.

### Design and implementation of weekly reporting through eIDSR

In addition to the DHIS2 platform, the MOH established the eIDSR platform in 2012 [12, 13], which was designed to use mobile phone technology to record and report surveillance data on 14 nationally notifiable diseases and 10 events of public health concern into the DHIS2 platform [20]. The eIDSR mobile phone functionality is based on Unstructured Supplementary Service Data (USSD) technology. Compared to other mobile phone reporting systems, the MOH selected USSD because of the following advantages: it is user-friendly by use of pop-up menus; provides accuracy through validity checks; is available on all mobile phone network providers; is free of charge to the end-user; has a fast two-way communication with the server; works on any mobile phone handset; and it does not require internet connection [21]. The eIDSR reporting portal is readily available to authorized users via the MOH mHealth short code [22]. As a surveillance platform, eIDSR can be configured to provide alerts when specific epidemic thresholds are exceeded in order to trigger appropriate investigations and response [12, 13].

Using the eIDSR reporting booklet [8, 22], each health facility compiles data for each epidemiological week (Monday to Sunday). The eIDSR users includes health facility IDSR focal persons, district and regional health teams, NMCP and the MOH Epidemiology Unit, and partners. In addition to the other 23 diseases, the malaria data collected weekly includes the: number of people tested for malaria by microscopy or RDT; number of patients with a positive test for malaria infection; and number of clinical malaria cases treated presumptively. Every Monday, health facility eIDSR focal persons submit the previous weeks’ data via the MOH mHealth USSD short code using existing mobile network providers. The weekly reports are stored in DHIS2 databases via the eIDSR server, and the system automatically sends feedback messages to the health facility to acknowledge the receipt of the weekly report. The deadline for submitting weekly reports is set for every Monday by 1530 h; reports submitted after this time are considered late reports. The system also sends text messages every Friday and Monday mornings to remind health care workers to send the weekly report.

**Fig. 1:**
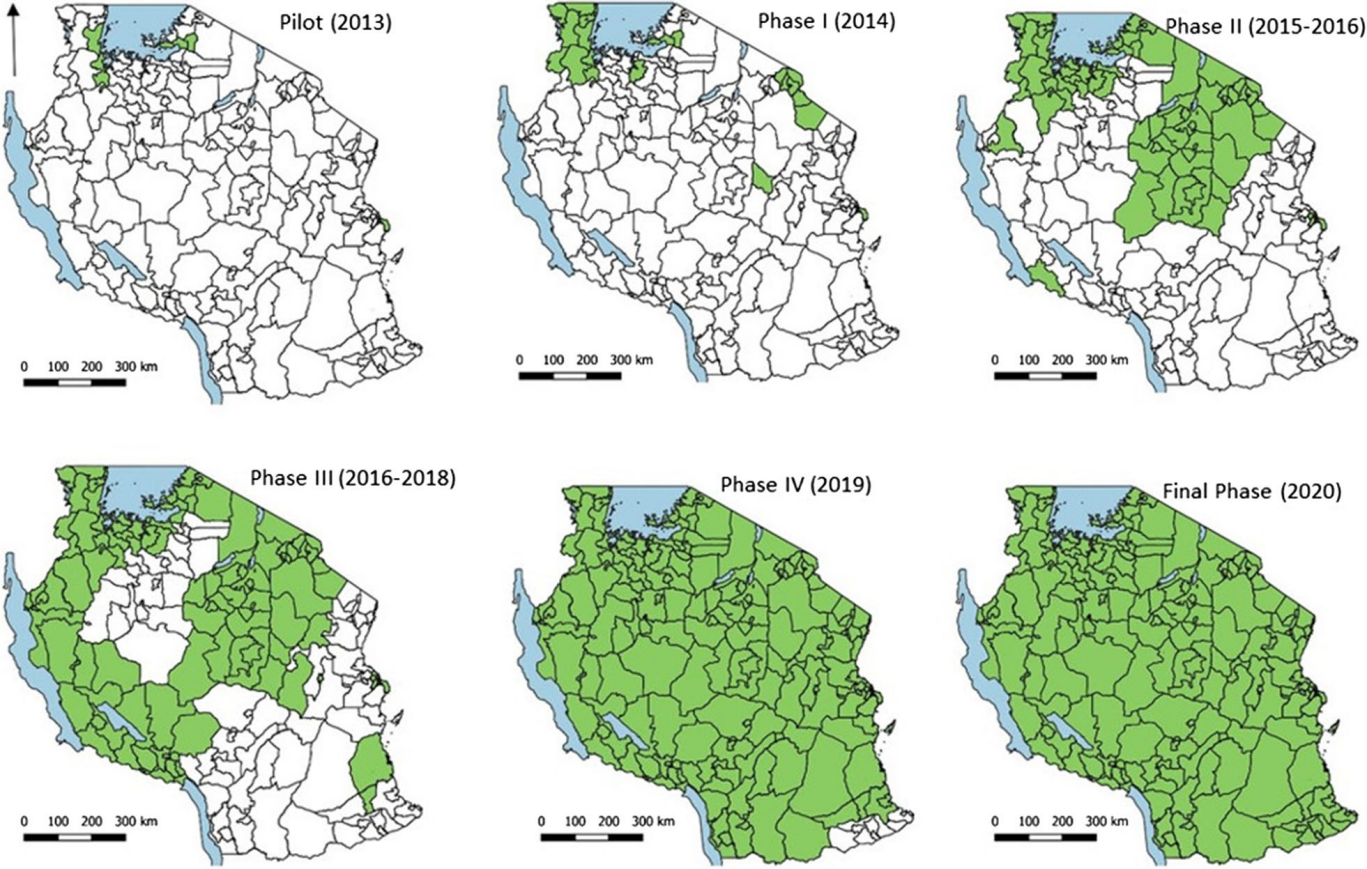
Phases of eIDSR scale-up in mainland Tanzania, 2013 to January 2020

The eIDSR platform was first piloted in 2013 in the mixed urban and semi-urban setting of Temeke Municipal Council in Dar es Salaam, comprising 67 public and private health facilities. This initial pilot enabled developers and administrators to study the system’s functionality, thus allowing for necessary adjustments before rolling it out nationally. Following a successful month-long implementation in Temeke, the pilot was extended to public and private health facilities in three Lake Zone districts: Bunda (Mara region), Chato (Mwanza region), and Muleba (Kagera region) [23]. After 3 months implementation of eIDSR in the four pilot districts, the system was further improved, and nation-wide roll-out in public and private health facilities commenced, with priority being given to Kagera region, which had the highest malaria burden nationally. From 2014 to 2020, the eIDSR platform was progressively scaled-up to reach all public and private health facilities in the 26 regions of mainland Tanzania (Fig. 1). In this manuscript, malaria data that are reported weekly are referred to as “weekly eIDSR”.

### Data analysis

The weekly eIDSR malaria data reported from 2013 to 2021 were downloaded from the DHIS2 database. Corresponding monthly DHIS2 data were downloaded for health facilities that were expected to send weekly eIDSR reports to enable like-for-like comparisons. Data were cleaned in Microsoft Excel, and analyses to compare weekly eIDSR and monthly DHIS2 data were conducted using Stata 15.0 (Stata Corporation, Texas, USA). Maps were developed using QGIS software version 3.0.3 (https://www.qgis.org/en/site/) [24]. Descriptive statistics were used to explore time trends of reporting, malaria testing and malaria cases.

Reporting completeness was defined as the proportion of reports submitted divided by the number of reports expected to be reported over as specified period (weekly, monthly, and annual). Reporting timeliness was defined as the proportion of reports submitted on time over the total number of reports expected to be reported. Time trends of malaria testing, malaria positivity and malaria cases (clinical and confirmed) were analysed. Testing proportion was defined as number of malaria diagnostic tests done divided by number of malaria diagnostic tests plus number of clinical malaria cases; positivity rate was defined as the number of positive malaria diagnostic tests divided by the total number of malaria diagnostic tests performed. The proportion of clinical cases was calculated by dividing the clinical cases and the total malaria cases (clinical and confirmed). Annualized malaria incidence per 1000 population for 2020 and 2021 was estimated by dividing the number of malaria cases and the mid-year population estimates based on the national census projections. Total malaria cases, incidence per 1000 and reporting rates were stratified by epidemiological strata as defined in the SMMSP [15], which were categorized as: “very low”; “low”; “moderate”; and “high” [14, 15]. Finally, consistency of weekly eIDSR and monthly DHIS2 data was investigated by comparing the reporting completeness, reporting timeliness, malaria cases and annualizes incidence by epidemiological strata and district for 2020 and 2021.

### Ethical considerations

Ethical clearance to undertake secondary analysis of surveillance data was granted by the National Institute of Medical Research (NIMR) with reference number: NIMR/HQ/R.8a/Vol.IX/3961.

## Results

### Phases of eIDSR implementation

Weekly malaria reporting through eIDSR was scaled-up gradually across the 26 regions of mainland Tanzania, with the initial pilot in four districts covering 182 public and private health facilities in 2013. Full roll-out was completed over the next 7 years, attaining 100% nation-wide coverage in 7,471 public and private health facilities by January 2020 (Fig. 1).

### Reporting completeness and timeliness

**Fig. 2:**
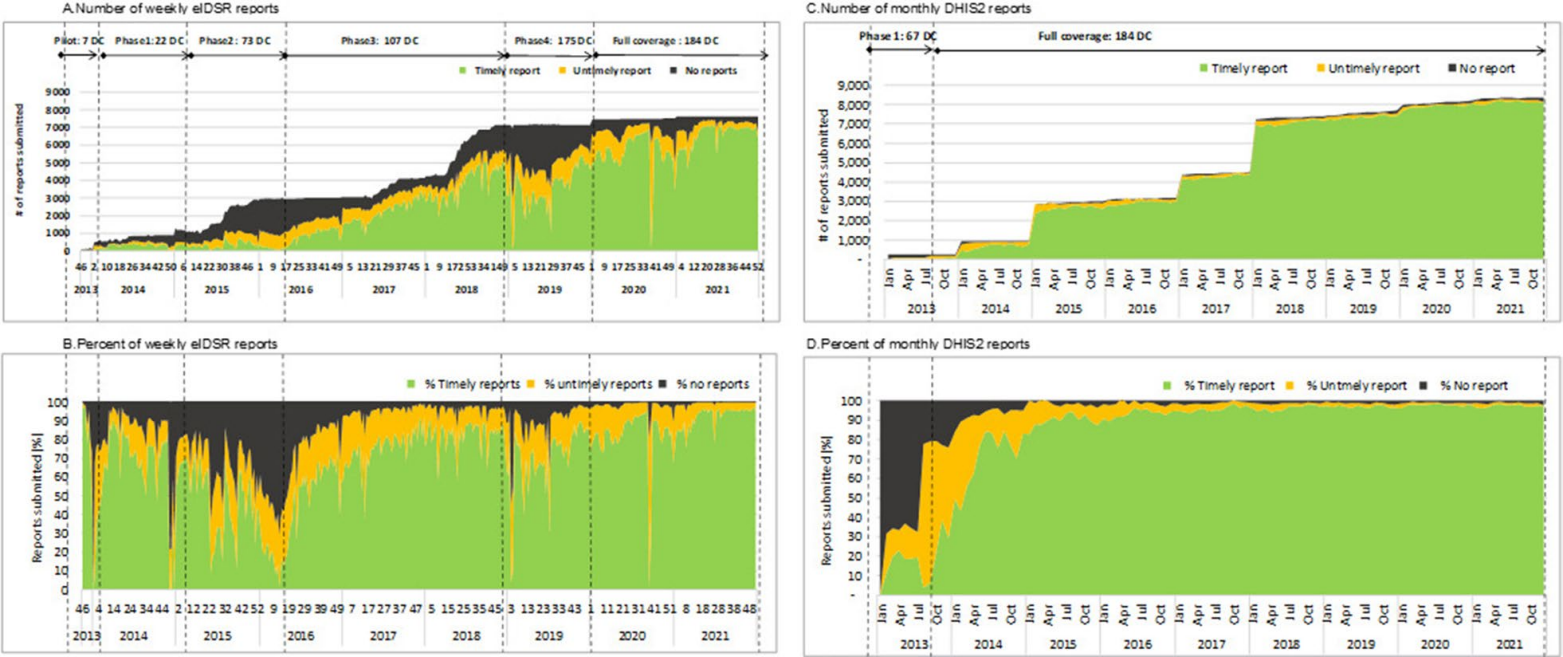
Time trends of weekly eIDSR and monthly DHIS2 reporting, 2013–2021. *DC* district councils

**Fig. 3:**
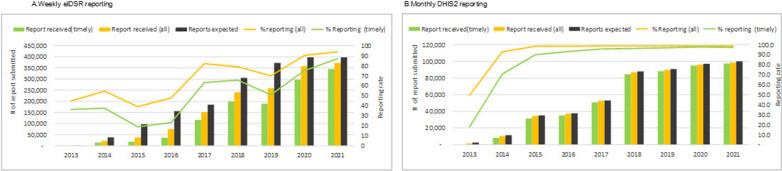
Time trends of weekly eIDSR and monthly DHIS2 reporting aggregated by year, 2013–2021

Figures 2 and 3 summarizes the time trends of reporting completeness and timeliness. Weekly eIDSR reporting completeness gradually increased over time; however, there were periodic cycles when reporting completeness and timeliness were lower (Figs. 2a, b and 3a). Apart from the initial pilot in 2013, monthly DHIS2 reporting completeness improved rapidly over time, and since 2017 both the overall reporting completeness and timeliness were consistently over 90% (Figs. 2c, d and 3b). After weekly eIDSR reporting attained nation-wide coverage, the overall reporting completeness was 90.2% in 2020 and 93.9% in 2021 (Fig. 3a). The reporting completeness from monthly DHIS2 data was 98.9% in 2020 and 98.7% in 2021 (Fig. 3b).

### Trends of malaria testing and positivity

**Fig. 4:**
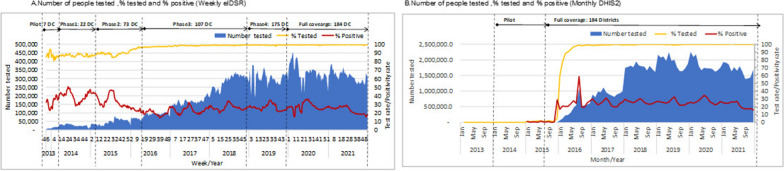
Time trends of weekly eIDSR and monthly DHIS2 malaria testing, 2013–2021. *DC* district councils

Figure 4 shows the trends of malaria testing and malaria positivity. Both weekly eIDSR and monthly DHIS2 data showed that malaria testing had increased over time (Fig. 4a and b). Overall, weekly eIDSR testing proportion increased from 87.0% in 2013 to 99.2% in 2021 (Fig. 4a). From 2016 onwards, the trends of malaria testing and malaria test positivity were similar for both weekly eIDSR and monthly DHIS2 (Fig. 4a and b).

### Trends of clinical and confirmed malaria cases

**Fig. 5:**
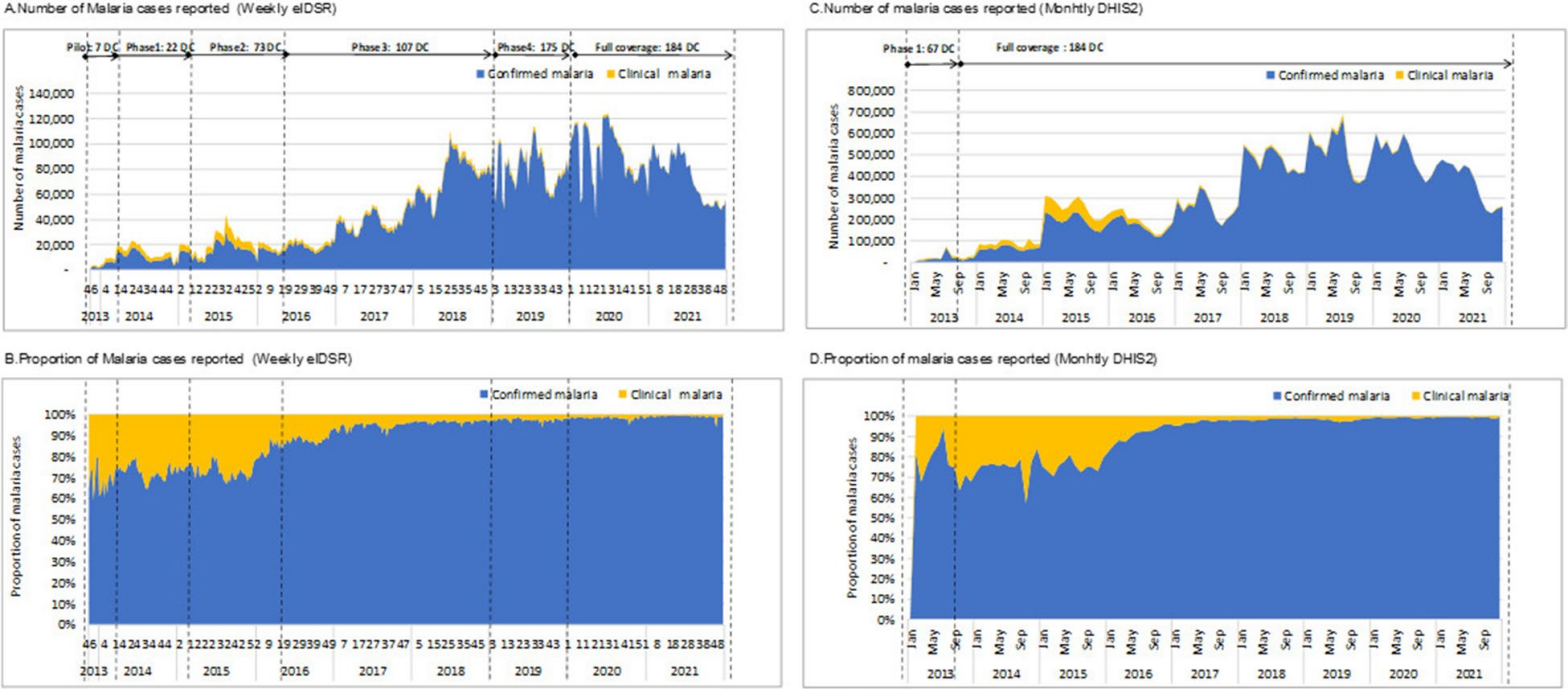
Number and proportion of suspected, and confirmed malaria cases, 2013–2021. *DC* district councils

Figure 5 shows the time trends of the number and proportion of clinical and confirmed malaria cases. Consistent with improvements in malaria testing, the proportion of clinical cases (treated presumptively without testing) reported through weekly eIDSR decreased from 30% before 2016 to 0.8% in 2021 (Fig. 5b). Similar declines in the number and proportion of clinical cases were observed for data reported through monthly DHIS2 (Fig. 5d).

### Comparison of weekly eIDSR and monthly DHIS2 reporting completeness, timeliness, malaria cases and malaria incidence, 2020 and 2021

**Fig. 6:**
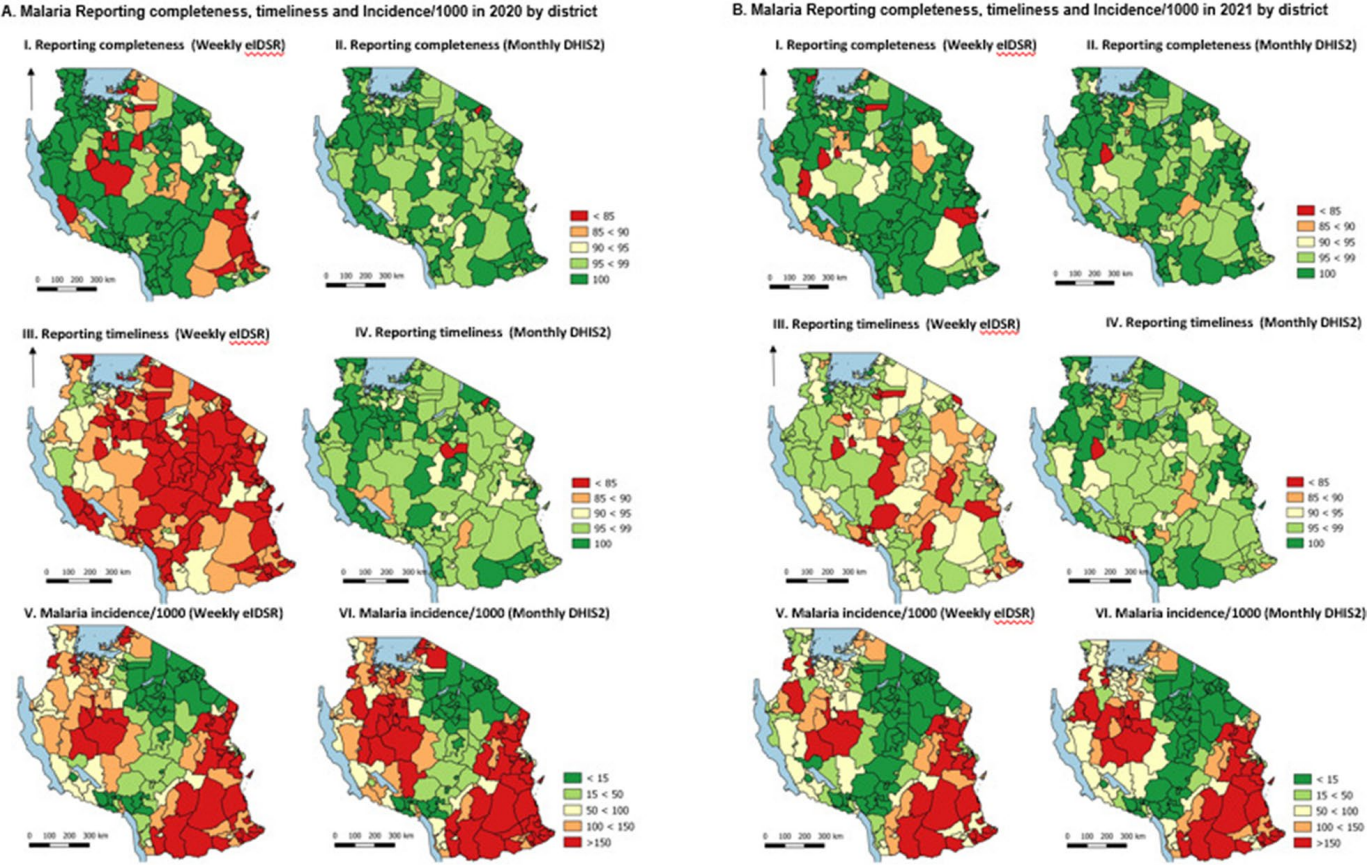
Comparison of malaria reporting completeness and incidence per 1,000 population by district 2020–2021

Comparisons between weekly eIDSR and monthly DHIS2 annualized reporting completeness and annualized malaria incidence in 2020 and 2021 are shown by epidemiological strata (Table 1) and by district (Fig. 6, map). In 2020, annualized malaria data reported through weekly eIDSR had 17.5% fewer cases compared to data reported through monthly DHIS2, with the annualized malaria incidence in 2020 being 87.0 per 1000 for weekly eIDSR data and 105.4 per 1000 for monthly DHIS2 data (Table 1). In 2021, annualized malaria data reported through weekly eIDSR had 12.4% fewer cases compared to monthly DHIS2. The annualized incidence in 2021 was 66.3 per 1000 for weekly eIDSR data and 75.7 per 1000 for monthly DHIS2 (Table 1). Annualized reporting rates varied by epidemiological strata (Table 1, Fig. 6). During 2020 and 2021, the “very low” epidemiological stratum had the highest overall reporting completeness and timeliness, while the annual incidence was similar across weekly and monthly data. For both 2020 and 2021, larger differences in malaria cases and incidence between the weekly eIDSR and monthly DHIS2 data were correlated with the lower weekly eIDSR reporting completeness rates (Fig. 6). Tarime District (2020) and Urambo district (2021) had the largest differences in reporting completeness of weekly and monthly data, while Mpimbwe District had the largest difference in incidence based on annualized weekly and monthly data for both 2020 and 2021 (Fig. 6).

**Table 1:** Comparison of monthly and weekly reporting trends, malaria cases and malaria annual incidence by malaria epidemiological strata, 2020–2021

Year	Epidemiological strata	Reporting completeness			Reporting timeliness			Total malaria cases (000 s)^a^			Annualized incidence per 1000 population		
		Weekly eIDSR	Monthly DHIS2	Percentage difference (%)	Weekly eIDSR	Monthly DHIS2	Percentage difference (%)	Weekly eIDSR	Monthly DHIS2	Percentage difference (%)	Weekly eIDSR	Monthly DHIS2	Percentage difference (%)
2020	Very low	94.1	98.3	4.2	78.1	97	19.7	64	64	0.5	6.3	6.6	4.6
	Low	90.8	98.3	7.7	73.8	96.5	23.5	383	462	17.0	26	32.8	20.8
	Moderate	89.1	99.7	10.6	72.2	98.8	26.9	1372	1663	17.5	96.5	117.2	17.7
	High	88.1	99.2	11.2	75.9	98.3	22.8	3177	3811	16.6	165	199.2	17.2
	Overall	90.2	98.9	8.8	75	97.7	23.3	4996	6000	16.7	87	105.4	17.5
2021	Very low	96.6	99.1	2.6	88.7	97.6	9.2	39	38	− 3.0	3.8	3.8	0.7
	Low	95.5	97.6	2.2	88.1	95.9	8.1	263	325	19.0	18.6	23.2	20.0
	Moderate	93	99.2	6.3	85.4	98.1	13	1049	1183	11.3	71.7	81.1	11.6
	High	91.7	99	7.3	86.1	97.6	11.9	2507	2840	11.7	127.1	144.5	12.0
	Overall	93.9	98.7	4.9	86.9	97.3	10.7	3858	4386	12.0	66.3	75.7	12.4

^a^Confirmed and clinical malaria cases

## Discussion

Following the WHO guidelines [7, 25], concerted efforts were made over the last decade to improve malaria surveillance in mainland Tanzania through weekly eIDSR and monthly DHIS2 systems. By 2020, mainland Tanzania had attained 100% coverage of weekly eIDSR reporting of malaria data in all public and private health facilities. The findings from this analysis suggest that reporting completeness and timeliness, malaria testing, and consistency of data reported through weekly eIDSR compared to monthly DHIS2 has improved over time, especially in “very low” epidemiological stratum. However, the analysis identified discrepancies in reporting completeness and incidence between weekly eIDSR and monthly DHIS2, suggesting areas for possible improvements in eIDSR reporting, particularly within the “low”, “moderate,” and “high” epidemiological strata.

The process undertaken by mainland Tanzania to improve malaria surveillance has involved several steps, including: (1) digitizing the routine monthly HMIS reporting tools through a web-based portal into DHIS2; (2) enhancing surveillance through weekly reporting of core malaria indicators using a mobile phone-based eIDSR platform; and (3) developing dashboards for data access and use. These tailored surveillance platforms can be expected to facilitate the targeting of malaria interventions to specific epidemiological settings in addition to enabling early detection and notification of changes in malaria in epidemic-prone settings [5, 25]. Based on the goals outlined in the SMMSP 2018–2020 [14], the current malaria surveillance system is now being expanded to include malaria case-based surveillance [i.e. case notification and household-based reactive case detection (RCD) and follow-up]. Household screening and treatment is an RCD approach that has been implemented effectively for nearly a decade in the malaria elimination setting of Zanzibar [26] and is now being considered in the “very low” epidemiologic stratum in mainland Tanzania [15].

Since the establishment of the electronic reporting platforms, reporting completeness and timeliness had improved considerably. As observed through the weekly eIDSR and monthly DHIS2 reporting, the proportion of confirmed malaria cases has increased markedly with a concomitant decrease in reporting of clinical cases that were treated presumptively. This improvement is largely due to implementation of malaria diagnosis and treatment guidelines that advocated for testing of all suspected malaria cases [25] and increased access to malaria testing through the nation-wide availability of malaria RDTs [27, 28], starting in 2013. Improved access to malaria diagnosis by microscopy or RDTs is an important aspect of malaria control, because it: enhances timely case detection and management; facilitates targeted and timely provision of commodities such as LLINs; and provides uniform case definitions for surveillance purposes. In addition, confirmed malaria cases form the basis for any case-based surveillance approaches.

The trends of malaria testing proportion observed in this analysis show evidence of the improvements in malaria testing undertaken in mainland Tanzania. A recent study showed that supply and availability of RDT had improved considerably from < 50% of health facilities in 2010 to 90% by 2016 [29] which is consistent with the testing proportion observed the analysis. However, there are concerns that stock outs of RDT influence testing rates [30] as evidenced by Bruxvoort et al. in a study done during the initial national roll-out of RDT in Tanzania in 2013 where approximately 50% of febrile patients were tested for malaria [31]. Therefore, stock outs of RDT may partly explain the fluctuations in testing overserved, especially in the weekly eIDSR data. On the other hand, the trends of malaria positivity shows conspicuous trough and peak patterns which are consistent with the seasonal transmission patterns with the mid-year peaks corresponding to the long rains from March to May (*Masika*) and the January peaks corresponding to the short rains from November to January (*Vuli*) [32]. However, despite these seasonal fluctuations, the rainfall patterns, temperatures, and humidity that characterize the tropical climate in mainland Tanzania support continuous malaria transmission year-round [3].

The monthly DHIS2 system attained national-wide coverage in 2014 and has remained the basis for routine reporting of malaria data and programmatic planning. In 2020, the overall malaria cases from monthly DHIS2 were consistent with numbers reported in the World Malaria Report 2021 [33]. Therefore, the monthly DHIS2 forms a reliable benchmark against which consistency of weekly eIDSR data could be compared. The comparisons of annualized reporting completeness and malaria incidence between weekly eIDSR and monthly DHIS2 data showed varying discrepancies by epidemiological strata. Notably, these differences were least in the “very low” epidemiological stratum, and the differences continued to decrease between 2020 and 2021. This improvement in consistency of data can be attributed to increased reporting completeness of weekly eIDSR in 2021 compared to 2020. The high level of concordance in reporting completeness, malaria cases and incidence between weekly eIDSR and monthly DHIS2 data in the “very low” epidemiological stratum is striking and suggests that the eIDSR platform provided accurate data that could readily be used as a malaria epidemic early detection system [30]. In addition, because eIDSR already has a built-in function for case notification, the platform could easily be expanded for CBS in the “very low” epidemiological stratum, as envisioned in the 2018–2020 SMMSP [15].

While this study was not an evaluation of the eIDSR platform, the system has several advantages: it is integrated into the routine HMIS at health facility level; uses a simple mobile phone reporting function; it has national-wide reach and is free of charge to each user at health facilities; data are reported into a common DHIS2 portal; and it provides “near real-time” access to data for decision making at all levels using various data visualization dashboards [8, 11]. In addition, eIDSR can be configured to provide alerts to inform the NMCP on when and where to trigger outbreak investigations once specific health facility-level thresholds are exceeded.

There are a number of potential limitations inherent in the surveillance systems described. Firstly, between 2020 and 2021, the study found differences between data reported through weekly eIDSR compared to monthly DHIS2. These differences were largely due to lower reporting completeness in weekly eIDSR, which suggests that further efforts are needed to improve weekly eIDSR reporting. In addition, it is also likely that the larger differences observed in low, moderate and high epidemiological strata were due to data quality issues affecting reporting in the health facilities with high disease burden, [34] since data extraction entails an additional workload and time. The differences may be partly attributed to the fact that the epidemiological week calendar year does not exactly align with the monthly calendar year. Secondly, there were periods of lower weekly eIDSR reporting completeness between 2020 and 2021 that possibly suggest there was an issue with eIDSR data transmission as opposed to failure by the health facilities in sending the weekly reports. Thirdly, it was not possible to estimate testing rate (number of malaria tests done divided by number of suspected cases) because the denominator was not one of the aggregate indicators reported routinely. Nonetheless, the testing proportion described in the analyses provides important evidence on how malaria testing has evolved and improved. Finally, since this study was not a formal evaluation of the eIDSR platform, it was not possible to fully explore eIDSR system performance based on this analysis. Therefore, given the limitations outlined, further investigations are required to evaluate the eIDSR system and undertake malaria data quality assessments to inform future improvements.

Strengthening surveillance systems by improving reporting rates and timeliness of reporting is crucial for malaria control and elimination efforts. The DHIS2 platform is being implemented in a number of malaria-endemic settings in sub-Saharan Africa and Asia [10, 35–39], with varied results. Lessons learned from prior DHIS2 country-wide rollouts suggest that designing and developing improved systems architecture that integrates electronic data collection and reporting will improve the quality and availability of data for decision-making [40–43]. To complement the WHO guidance for analysis and use of health facility data [42, 43], Tanzania has developed a malaria dashboard within DHIS2 in order to increase data use [18]. To increase malaria data demand and use, starting in 2017, the NMCP introduced regional and zonal data review meetings at national and subnational levels [44]. To maximize the demand and use of malaria surveillance data to target interventions more effectively in Tanzania, further work is needed to: assess the utility and impact of malaria data dashboards; establish appropriate epidemic thresholds and alerts in the eIDSR weekly reporting; and deploy case notification and RCD in the “very low” epidemiological stratum.

## Conclusions

These findings suggest that reporting through weekly eIDSR had improved steadily over time; however, discrepancy in weekly eIDSRs’ reporting completeness, malaria cases and malaria incidence for 2020 and 2021 compared to monthly DHIS2 data suggest that further improvements are needed. Nonetheless, the concordance between weekly eIDSR and monthly DHIS2 data in the “very low” epidemiological stratum suggests that eIDSR system could reliably be expanded as a malaria early epidemic detection system (MEEDS), and to include case notification, RCD and follow-up. In addition, it can be expected that improvements in reporting rates and timeliness of weekly eIDSR data will improve the overall quality of malaria data and allow the NMCP to more effectively tailor its programmatic activities to help achieve national and global malaria elimination goals.

## Data Availability

The datasets used and /or analysed during the current study are available from the corresponding author on reasonable request.

## Abbreviations

ACT: Artemisinin-based combination therapy
ACD: Active case detection
CBS: Case-based surveillance
DHIS: District health information system
eIDSR: Electronic Integrated Disease Surveillance and Response
HMIS: Health management information system
ITN: Insecticide-treated nets
IRS: Indoor residual spraying
LLIN: Long-lasting insecticidal net
MEEDS: Malaria epidemic early detection system
MOH: Ministry of health
NMCP: National malaria control programme
PMI: President’s malaria initiative
RCD: Reactive case detection
RDT: Rapid diagnostic test
USAID: United States Agency for International Development
USSD: Unstructured supplementary service data
WHO: World Health Organization
